# *In Vivo* Pharmacokinetic/Pharmacodynamic Profiles of Danofloxacin in Rabbits Infected With *Salmonella typhimurium* After Oral Administration

**DOI:** 10.3389/fphar.2018.00391

**Published:** 2018-04-17

**Authors:** Xia Xiao, Lin Pei, Li-Jie Jiang, Wei-Xuan Lan, Jia-Yu Xiao, Yon-Jia Jiang, Zhi-Qiang Wang

**Affiliations:** ^1^College of Veterinary Medicine, Yangzhou University, Yangzhou, China; ^2^Jiangsu Co-innovation Center for Prevention and Control of Important Animal Infectious Diseases and Zoonoses, Yangzhou, China; ^3^Institute of Agricultural Science and Technology Development, Yangzhou, China

**Keywords:** danofloxacin, *S. typhimurium*, PK/PD modeling, parameter magnitude, rabbit

## Abstract

*Salmonella typhimurium* is a highly transmissible pathogen in rabbits that causes significant losses. Danofloxacin shows excellent efficacy against *S. typhimurium* infections. However, there are few reports of the pharmacokinetic/pharmacodynamic (PK/PD) modeling of danofloxacin against this pathogen. The aim of this study was to evaluate the *in vivo* PK/PD relationship of danofloxacin in rabbits infected with *S. typhimurium.* We used the reduction of bacterial burden in the blood, liver, spleen, and lung as the target PD endpoints, and determined the PK/PD indexes that best correlated with the efficacy and its corresponding magnitude. Danofloxacin was administrated orally to experimentally *S. typhimurium*-infected rabbits once daily for three successive days. The concentrations of danofloxacin in the serum and the bacterial burden in the blood, liver, spleen, and lung were determined. The PK/PD relationships of danofloxacin against *S. typhimurium* were evaluated using a Sigmoid E_max_ model. The results showed that the area under the concentration-time curve from 0 to 24 h/minimum inhibitory concentration (AUC_24 h_/MIC) ratio correlated well with the *in vivo* antibacterial effectiveness in different organs, with an r^2^ of 0.8971, 0.9186, 0.9581, and 0.8708 in the blood, liver, spleen, and lung, respectively. The AUC_24 h_/MIC ratios for the bactericidal effect (3 × Log_10_ colony forming units/mL reductions) were 121.30, 354.28, 216.64, and 228.66 in the blood, liver, spleen, and lung, respectively, indicating that the *in vivo* effectiveness of danofloxacin against *S. typhimurium* using bacterial reduction in different organs as PD endpoints was not identical. This study illustrated that the selection of the target organ for bacterial reduction determination had little effect on best PK/PD parameter determination, but is critical for parameter magnitude calculation in antimicrobial PK/PD modeling, and furthermore, has an impact on the rational dosage optimization process.

## Introduction

*Salmonella typhimurium* is associated with 1 million human deaths annually (Preethi et al., 2017). It is the most important serotype of *Salmonella* that is transmitted from animals to humans, causing a marked threat to public health (Preethi et al., 2017). Rabbits are an important source of meat and demand is increasing because of its nutritional and dietary properties (Kohler et al., 2008; Dalle Zotte and Szendro, 2011). *S. typhimurium* is a common serovar causing abortion or systemic infection in rabbits at different stages, especially before and during weaning (Camarda et al., 2013). Infection is mainly controlled using antimicrobials. Danofloxacin, the third generation of fluoroquinolones, is applied solely for veterinary use and displays excellent antimicrobial activity against both gram-negative and gram-positive bacteria (Shojaee Aliabadi and Lees, 2003). However, because of the development of resistance in bacteria, its efficacy is decreasing. It is generally considered that inappropriate use of antibacterials is the main reason for resistance development (Marshall and Levy, 2011). Thus, to preserve the effectiveness of danofloxacin, the dose regimen should be optimized to maximize the therapeutic effect and minimize the emergence of resistance (Li et al., 2017). A rational antibacterial dose regimen should be based on a good understanding of the pharmacokinetic and pharmacodynamic (PK/PD) relationship that bridges the gap between the PKs of an antibacterial in the target animal species and the PDs on the target bacteria species (Andes and Craig, 2002; Paladino and Callen, 2003; Tuntland et al., 2014). The *ex vivo* PK/PD model of danofloxacin has been studied for several pathogens such as *Escherichia coli*, *Mannheimia haemolytica*, and *Staphylococcus aureus* in calves, sheep, goats, camels, chickens, and turkeys (Aliabadi et al., 2003a,b; Shojaee Aliabadi and Lees, 2003; Haritova et al., 2006; Serrano-Rodriguez et al., 2017). *In vivo* PK/PD modeling was also conducted in chickens against *Mycoplasma gallisepticum* (Zhang et al., 2017). However, neither *ex vivo* nor *in vivo* PK/PD modeling of danofloxacin against *S. typhimurium* has been established.

Bacterial diseases may cause different bacterial burdens in different organs. However, PD evaluation in PK/PD modeling usually focuses on bacterial reduction in just one organ. Whether bacterial reduction in one organ can represent the efficacy of the drug in the host is unknown. Some studies reported diverse antibacterial actions in different organs after drug administration (Haritova et al., 2011; Swanson et al., 2015). The phenomenon indicated that the PK/PD surrogate value of an antimicrobial for the same effect might not be identical using bacterial reduction in different organs as PD endpoints.

Thus, in the present study, *in vivo* PK/PD modeling of danofloxacin against *S. typhimurium* was developed using the reductions of the bacterial burden in the blood, liver, spleen, and lung as the PD endpoints. The PK/PD indexes that best correlated with efficacy, and its corresponding magnitude, were determined.

## Materials and Methods

### Organisms, Chemicals, and Animals

The virulent *S. typhimurium* strain, HJ0310J was kindly supplied by Professor Da-Xing Peng (College of Veterinary Medicine, Yangzhou University, Yangzhou, Jiangsu Province, China). Danofloxacin mesylate (98%) was purchased from Yuanye Biotechnology, Co. Ltd. (Shanghai, China). The culture medium used in this experiment was purchased from Hope Biol-Technology, Co. Ltd. (Qingdao, China). One hundred and fifteen *S. typhimurium*-free New Zealand rabbits aged 5–6 weeks and weighing 0.8–1.2 kg were used in this study. Animals were housed in a clean environment for 1 week before the study, with free access to antibiotic-free feed and water. All the protocols were carried out in accordance with the guidelines of American Association for Accreditation of Laboratory Animal Care, Institute of Laboratory Animal Research, Commission on Life Sciences. The study was approved by the Animal Experiments Ethics Committee at Yangzhou University, with permission number SYXK(Su) IACUC 2017-0044.

### *In Vitro* Pharmacodynamics Study

The minimum inhibitory concentration (MIC) of danofloxacin against *S. typhimurium* and the standard organism *Salmonella enterica* subsp. *enterica* (ex Kauffmann and Edwards) Le Minor and Popoff serovar Typhimurium (ATCC 14028), cultivated in Mueller-Hinton (MH) broth, was evaluated using the micro-dilution method, according to the Clinical and Laboratory Standards Institute (CLSI, 2013). The MIC in rabbit serum was also determined using the micro-dilution method according to a previous report (Zhao et al., 2014). The minimum bactericidal concentration (MBC) in artificial medium and in rabbit serum was measured according to a previous report (Lei et al., 2017). Briefly, a 100-μL aliquot of suspensions from the MIC procedure were diluted and dropped onto MH agar plates. The bacteria were counted after incubation at 37°C for 48 h. The lowest concentration of danofloxacin that killed 99.9% of the bacteria was defined as the MBC. The mutant prevention concentration (MPC) was determined according to a previous report (Gebru et al., 2012). The organism was cultured in MH broth for 24 h at 37°C, centrifuged at 3000 × *g* for 10 min, and re-suspended in MH broth to yield a concentration of ∼10^10^ colony forming units (cfu)/mL (confirmed through serial dilution and plating of 100 μL samples on MH agar). Then, ∼10^10^ cfu of microorganisms was spread on MH agar including serial concentrations of danofloxacin (1–32 × the MIC) and then incubated at 37°C for 96 h. The MPC was the lowest concentration that inhibited bacterial growth (Blondeau et al., 2001). The *in vitro* time kill curves of danofloxacin against *S. typhimurium* were determined at concentrations of 0, 0.5, 1, 2, 4, 8× the MIC, with an initial inoculum of ∼10^6^ cfu/mL. All experiments were performed in triplicate.

### *Salmonella typhimurium* Infection Model

*Salmonella typhimurium* is a major cause of systemic infection in rabbits. Thus, an *S. typhimurium* infection model was used in this study. Briefly, strain HJ0310J was grown in broth overnight, and then centrifuged and re-suspended in commercially available saline. Animals were divided into four groups, five rabbits per group, and injected with 0.5 mL of saline containing 0, 10^7^, 10^8^, and 10^9^ cfu/mL of bacteria through the ear marginal vein. The same amounts of bacteria were injected 12 h later. Twelve hours after the last bacterial injection, the clinical symptoms, anatomy, and pathological changes were monitored. And then, animals were sacrificed by a lethal intravenous injection of beuthanasia (0.3 mL/kg) after anesthesia with ketamine- Xylazine. Bacterial loads in the blood, liver, spleen, and lung were estimated via dilution of tissue homogenates and plating onto Salmonella Shigella agar. The colonies were counted after incubation at 37°C for 24 h. The limit of detection was 2 × 10^3^ cfu/mL or 2 × 10^3^ cfu/g. The mortality rate was monitored until 84 h after the last bacterial inoculation using another four groups of animals (five rabbits per group).

### Pharmacokinetics of Danofloxacin in the Infected Rabbits

Thirty infected rabbits were divided into three groups randomly and treated with a single dose of 1, 10, or 30 mg/kg body weight (b.w.) danofloxacin via gavage. An aliquot of 0.5 mL of blood was collected from ear vein at 0.25, 0.5, 0.75, 1, 2, 4, 8, 12, and 24 h post-drug administration. Serum was obtained through centrifuging the samples for 10 min at 3000 × *g*. High performance liquid chromatography with a fluorescence detector was used to determine the danofloxacin concentrations in serum, as described previously, with some modifications (Garcia et al., 2000; Goudah and Mouneir, 2009). Briefly, a 1-mL aliquot of trichloromethane was added to 200 μL of serum sample, vortexed for 5 min, and centrifuged at 12000 × *g* for 10 min. The supernatant was transferred, and the residue was re-extracted. The extracts were combined and evaporated to dryness under a gentle stream of nitrogen at 40°C. The residue was dissolved in 200 μL mobile phase containing phosphate buffer and acetonitrile (83:17, V/V, pH 3). Isocratic elution was used. The excitation and detection wavelengths were 280 and 450 nm, respectively. Good sensitivity and chromatographic behavior was achieved under this condition (**Supplementary Figure S1**). The recovery rate was 95.43–105.2%, and the coefficients of variation of inter-assay and intra-assay precision were lower than 10%. The limit of quantification (LOQ) was 0.02 μg/mL and the limit of determination (LOD) was 0.01 μg/mL. The pharmacokinetic data for danofloxacin were analyzed using the non-compartmental method in the WinNonlin software version 6.1 (Pharsight, St. Louis, MO, United States).

### Effectiveness of Danofloxacin in the Infection Model

To evaluate the *in vivo* effectiveness of danofloxacin, infected rabbits were treated via gavage once daily for 3 successive days with 0.85% NaCl (control) or 1, 2.5, 5, 7.5, 10, 15, 20, and 30 mg/kg b.w. of danofloxacin (five animals per group). Treatment started at 12 h post-infection. At 24 h after the last dose, the animals were euthanized, and then their blood, livers, lungs, and spleens were sampled sterilely. The samples were homogenized and diluted with 0.85% NaCl. The bacterial loading in each organ was determined via plating dilutions onto Salmonella Shigella agar and counting the colonies after incubation at 37°C for 24 h.

### Pharmacokinetics and Pharmacodynamics Analysis

Fluoroquinolones such as danofloxacin are concentration-dependent drugs and the surrogate marker area under the concentration-time curve from 0 to 24 h/minimum inhibitory concentration (AUC_24 h_/MIC) ratio is the best PK/PD parameter. Therefore, as a surrogate marker of danofloxacin’s effectiveness, the AUC_24 h_/MIC ratio was determined according to the *in vitro* MIC values and the PK parameters derived from three danofloxacin administrations via gavage. The effectiveness of danofloxacin was expressed as the bacterial reduction after treatment compared with that before treatment in each organ. The *in vivo* PK/PD relationships of danofloxacin in each organ were simulated using a sigmoid E_max_ model in the WinNonlin software (version 6.1; Pharsight) using the following equation:

$$E=E_{0}+\frac{E_{\max}\times C_{e}^{N}}{{EC}_{50}^{N}+C_{e}^{N}}$$

Where, E_0_ is the change in log_10_ cfu/mL or log_10_ cfu/g in the control sample (absence of danofloxacin). E_max_ is the difference in effect between the greatest amount of growth (as seen for the growth control, E_0_) and the greatest amount of killing. C_e_ is the AUC_24 h_/MIC in the effect compartment. EC_50_ is the AUC_24 h_/MIC value producing a 50% reduction in bacterial counts, and N is the Hill coefficient that describes the steepness of the AUC_24 h_/MIC-effect curve (Burton et al., 2005).

### Statistical Analysis

The pharmacokinetic parameters of danofloxacin were presented as the mean ± SD. They were computed with the WinNonlin software (version 6.1; Pharsight). The same software was used for curve fitting to determine the relationship between the AUC_24 h_/MIC ratios and the efficacy of danofloxacin. The GraphPad Prism software was used for time kill curve fitting. Other data were analyzed using SPSS (v.20) (SPSS Incorporated, Chicago, IL, United States). *P* < 0.05 was regarded as statistically significant.

## Results

### *In Vitro* Pharmacodynamics

**FIGURE 1:**
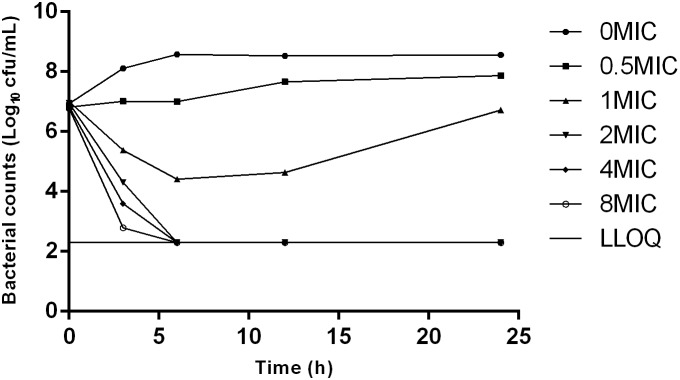
Time-killing curves of danofloxacin against *Salmonella typhimurium* in Mueller-Hinton (MH) broth with concentrations of danoflocaxin between 0 and 8× the minimum inhibitory concentration (MIC).

The MIC and MBC of danofloxacin against *S. typhimurium* HJ0310J in MH broth were 0.03 and 0.06 μg/mL, while the corresponding values in rabbit serum were twice as high, at 0.06 and 0.12 μg/mL; respectively. The MPC in medium was eight times higher than the MIC, with a value of 0.25 μg/mL. The time-killing curve showed that with increasing amounts of the drug, the antibacterial effect was enhanced. Maximal killing was observed at 2× the MIC of danofloxacin (**Figure 1**).

### *S. typhimurium* Infection Model

**FIGURE 2:**
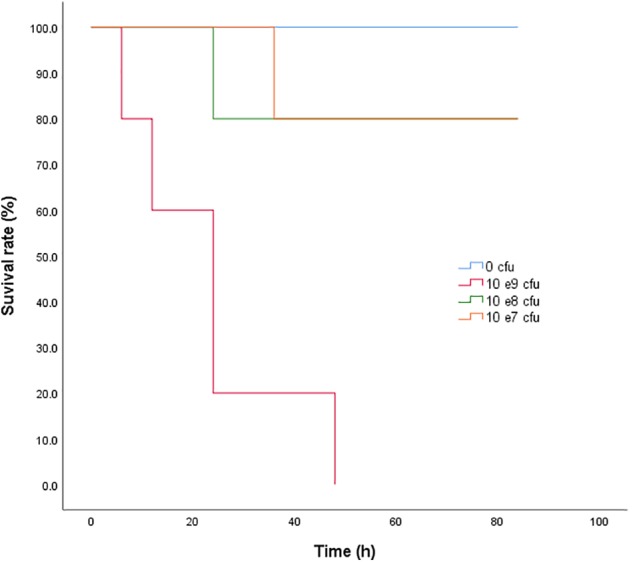
The survival rate of rabbits 84 h after the last bacterial inoculation.

Neither clinical signs nor anatomopathological lesions were observed in the control animals. The bacteriological assay was also negative in the control rabbits. Clinical symptoms, such as depression, decreased feeding, diarrhea, and fever, were observed in the other three infected groups. The symptoms became worse as the bacterial inoculation increased. After dissection, the bowel, spleen, and gallbladder were swollen, and there were bleeding spots in the duodenum. The survival curve of rabbits receiving 0, 0.5 × 10^7^, 0.5 × 10^8^, and 0.5 × 10^9^ cfu of *S. typhimurium* are shown in **Figure 2**. The corresponding survival rates of the four groups were 100, 80, 80, and 0% respectively. The bacterial loads in the blood, liver, spleen, and lung at 12 h after the last inoculation of ∼0.5 × 10^8^ cfu *S. typhimurium* HJ0310J were 6.51 ± 0.32, 6.56 ± 0.26, 7.73 ± 0.18, and 7.32 ± 0.32 log_10_ cfu/g, respectively. As a result, a inoculate of ∼0.5 × 10^8^ cfu was chosen for the infection model.

### Pharmacokinetics of Danofloxacin in Infected Rabbits

**FIGURE 3:**
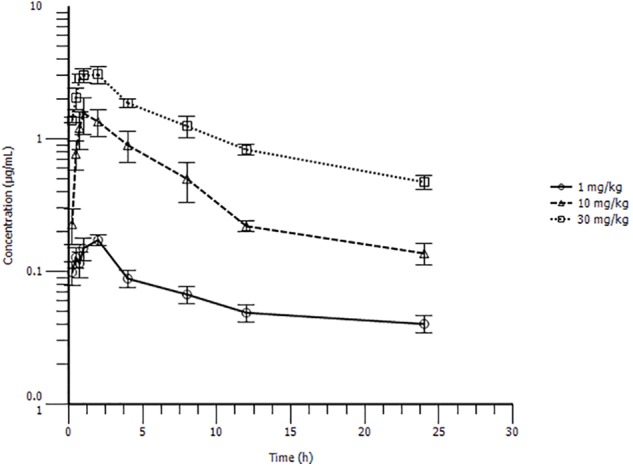
Serum drug concentration-time profile of danofloxacin after oral administration at 1, 10, or 30 mg/kg body weight (b.w.) in *S. typhimurium* infected rabbits (*n* = 10/time point).

The PK profiles of danofloxacin and the main PK parameters are presented in **Figure 3** and **Table 1**. The time of peak serum concentration (T_max_), elimination half-life (T_1/2β_), and clearance divided by bioavailability (CL_β_/F) for the three different doses showed little difference. However, the maximum serum concentration (C_max_) (0.18, 1.80, and 3.49 μg/mL) and AUC_24 h_ (1.55, 11.70, and 28.10 μg h/mL) of the three doses increased in a concentration-dependent manner. A significant correlation between dose and C_max_ or AUC_24 h_ was observed, with r^2^ values equal to 0.9592 and 0.9935, respectively. Thus, the AUC_24 h_ of 2.5, 5, 7.5, 15, and 20 mg/kg b.w. doses could be deduced according to a linear relationship.

**Table 1:** Pharmacokinetic parameters of danofloxacin in serum following oral administration to *Salmonella typhimurium-*infected rabbits (*n* = 10/group).

Parameter	1 mg/kg Mean ± *SD*	10 mg/kg Mean ± *SD*	30 mg/kg Mean ± *SD*
C_max_	0.18 ± 0.04	1.80 ± 0.98	3.49 ± 0.91
T_max_	1.60 ± 0.55	1.80 ± 1.30	1.40 ± 0.55
T_1/2β_	15.30 ± 6.09	14.01 ± 9.34	11.38 ± 3.16
AUC_24 h_	1.55 ± 0.39	11.70 ± 4.47	28.10 ± 3.27
CL_β_/F	0.45 ± 0.11	0.76 ± 0.30	0.84 ± 0.09
Vz/F	9.36 ± 3.79	14.67 ± 10.00	13.65 ± 3.57


*C_*max*_ (μg/mL), maximum serum concentration; T_*max*_ (h), time of maximum serum concentration; T_½β_, elimination half-life; AUC_*24 h*_ (μgh/mL)_,_ 24 h area under serum concentration-time curve; F, bioavailability; CL*_β_*/F (L/h/kg), clearance divided by bioavailability; V_*z*_/F (L/kg), volume of distribution scaled by bioavailability.*

### *In Vivo* Pharmacokinetic and Pharmacodynamic Profiles of Danofloxacin

**FIGURE 4:**
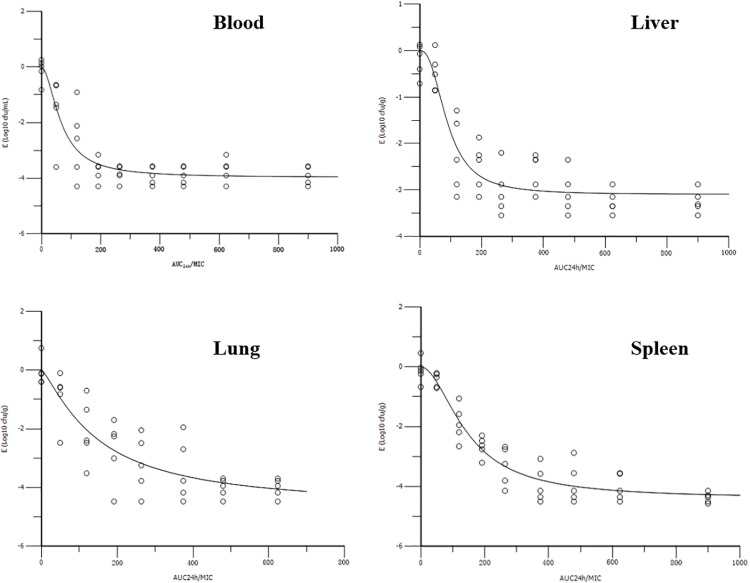
Sigmoid E_max_ relationships between danofloxacin’s antibacterial effect in the blood, liver, spleen, and lung [E, Log_10_ colony forming units (cfu)/mL or Log_10_ cfu/g] and the *in vivo* area under the concentration-time curve from 0 to 24 h (AUC_24 h_)/minimum inhibitory concentration (MIC) ratio in rabbits.

The AUC_24 h_/MIC ratios for doses of 0, 1, 2.5, 5, 7.5, 10, 15, 20, and 30 mg/kg were 0, 76.42, 119.60, 191.56, 263.54, 335.50, 479.44, 623.38, and 911.24, respectively. The profiles of the sigmoid E_max_ model describing the relationship between AUC_24 h_/MIC and antibacterial effectiveness in each organ are presented in **Figure 4**. The surrogate AUC_24 h_/MIC correlated well with effectiveness in each organ, with r^2^ values of 0.8971, 0.9186, 0.9581, and 0.8708 for blood, liver, spleen, and lung, respectively. The AUC_24 h_/MIC value for the bactericidal effect in the blood, liver, spleen, and lung were 121.30, 354.28, 216.64, and 228.66, respectively (**Table 2**). The slopes were 1.86, 2.47, 1.90, and 1.26 for the blood, liver, spleen, and lung, respectively. Thus, the *in vivo* effectiveness of danofloxacin against *S. typhimurium* in different organs varied.

**Table 2:** Pharmacodynamic analysis of danofloxacin in infected rabbits targeting the blood, liver, spleen, and lung.

Parameter (units)	Blood	Liver	Spleen	Lung
E_max_ (Log10 cfu/mL)	3.98	3.1	4.41	4.74
E_0_ (Log_10_ cfu/mL)	0.00	0.00	0.00	0.01
EC_50_	66.54	89.84	145.66	148.18
AUC_24 h_/MIC for a 3 Log10 cfu/mL reduction	121.30	354.28	216.64	228.66
Slope (N)	1.86	2.47	1.90	1.26


*AUC_*24h*_/MIC: area under the concentration-time curve from 0 to 24 h/minimum inhibitory concentration ratio.*

## Discussion

Fluoroquinolones are critical important antibiotics for human and animal health, to preserve the effectiveness of this kind of drugs, PK/PD modeling, especially *in vivo* PK/PD modeling, should be investigated to design a rational dosage (Craig, 1998; Xiao et al., 2015; Andes and Lepak, 2017). In the present study, *in vivo* PK/PD modeling of danofloxacin against *S. typhimurium* in rabbits was conducted and a series of interesting findings emerged.

The MIC and MBC for danofloxacin against *S. typhimurium* in rabbit serum were twice those in artificial broth, which could be explained by the protein binding ratio of danofloxacin in serum (Qu et al., 2015) and the differences of matrix. In accordance with previous reports, in which the MPC was eight times higher than the MIC (Lorenzutti et al., 2017), the ratio of MPC/MIC in this study was 8, indicating that the mutant selection window is wide. The *in vitro* time-killing curve of danofloxacin showed concentration dependence against *S. typhimurium*, which was similar to a previous publication (Lei et al., 2017).

This is the first report of danofloxacin pharmacokinetics in *S. typhimurium*-infected rabbits after oral administration. The CL/F of danofloxacin for three doses, ranging from 0.45 to 0.84 L/h/kg, showed little difference compared with a previous report, where the CL was 0.76 L/h/kg after intravenous injection of 6 mg/kg danofloxacin in healthy rabbits (Fernandez-Varon et al., 2007). However, the T_1/2β_ values in this study (15.30, 14.01, 11.38 h for 1, 10, 30 mg/kg b.w.) were much longer than those in healthy rabbits (4.88, 6.70, and 8.2 h for intravenous, intramuscular, and subcutaneous administration, respectively) (Fernandez-Varon et al., 2007). The longer T_1/2β_ in sick rabbits might reflect injury to their liver and kidneys, which agreed with the results of a previous publication (Xiao et al., 2014). The results showed that *S. typhimurium* infection affects the elimination half-life of danofloxacin in rabbits and may further affect the PK/PD modeling results. The dose proportionality of danofloxacin pharmacokinetics in the range of 1 mg/kg to 30 mg/kg was described in the *S. typhimurium*-infected rabbits. The dose and AUC_24 h_ or C_max_ showed significant correlations, which was in accordance with a previous report (Zhang et al., 2017).

To the best of our knowledge, this is the first report of *in vivo* PK/PD modeling of danofloxacin against *S. typhimurium*. Targeting the bacterial burden in different organs, the PK/PD surrogate AUC_24 h_/MIC showed a significant correlation with the *in vivo* antibacterial effects of danofloxacin against *S. typhimurium*, with an r^2^ value greater than 0.8708. However, the value of the PK/PD parameter, AUC_24 h_/MIC, to attain a bactericidal effect, was different in each organ. It was reported that the concentration of danofloxacin in the lung was many times higher than that in plasma (Yang et al., 2015; Zhang et al., 2017) and the V_z_ (ranging from 9.36 to 13.65 L/kg) in this study, indicating that danofloxacin can accumulate in tissues such as the lung, spleen, and liver. The AUC_24 h_/MIC value for the bactericidal effect in the spleen and lung were almost twice those in the blood (216.64, 228.66, and 121.30 for the spleen, lung, and blood, respectively). One explanation could be that the initial bacterial loading in the spleen and lung was 10 times higher than that in the blood. It was reported previously that the initial bacterial loading seriously affects the PK/PD parameter values to attain a certain efficacy (Guo et al., 2016). However, the AUC_24 h_/MIC value for the bactericidal effect in the liver (354.28) was higher than that in spleen and lung, and almost three times that in the blood. In addition, the initial bacterial load in the liver was the same as that in blood. The infection in this study was a severe model of sepsis based on a high load inoculation by the intravenous route. In addition, it was reported that the clearance of bacteria reflected the cooperation of antimicrobial and immune responses in the host (Ankomah and Levin, 2014). The spleen and liver play an important function (as filters of bacteria from the bloodstream) in the clearance of bacteria during bloodstream infection. The host defense mechanisms, especially hepatic clearance, contribute greatly to containing bacteremia (Christaki and Giamarellos-Bourboulis, 2014). Thus, the AUC_24 h_/MIC value for the bactericidal effect in the liver was much higher than that in blood and other organs. Similar results were reported in previous publications, where the bactericidal action of clofazimine against *M. tuberculosis* in the spleen was much stronger than that in the lung (Swanson et al., 2015); meanwhile, the re-isolation rate of bacteria from the liver was much higher than that from spleen after the administration of a series of doses of enrofloxacin in *E. coli*-infected poultry (Haritova et al., 2011).

To date, many reports focusing on the PK/PD relationship of fluoroquinolones have been published. Toutain reported that an AUC_24 h_/MIC of 125 for fluoroquinolones was usually used as a threshold for ideal therapeutic outcome against gram-negative microbes (Toutain et al., 2002). The PK/PD results in this study for the bacterial burden in the blood was similar to this threshold. Similarly, the AUC_24 h_/MIC value for danofloxacin was close to 125 when deduced from *in vivo* PK/PD modeling. For example, the value of AUC_24 h_/MIC for 3 Log_10_cfu/mL reduction was 97.98 h in chicken against *M. gallisepticum* (Zhang et al., 2017). However, the value of AUC_24 h_/MIC of danofloxacin was lower than 125 when deduced from *ex vivo* PK/PD modeling. For example, the AUC_24 h_/MIC value was 6.73 in turkeys against *E. coli* (Haritova et al., 2006); 33.5 h in calves against *Mannheimia haemolytica* (Shojaee Aliabadi and Lees, 2003); and 21.2 h in camels against *E. coli* (Aliabadi et al., 2003a). This phenomenon was also reported for other drugs, such as valnemulin (Xiao et al., 2015). The possible reasons were elaborated in our previous study (Xiao et al., 2015). These results further confirmed that *in vivo* PK/PD modeling has great advantages compared with *ex vivo* PK/PD modeling, and is more suitable to determine antibacterial efficiency predictors. However, the PK/PD parameter for bactericidal activity in this study targeted at the bacterial burden in the liver, spleen, and lung were much higher than 125, taking into consideration the immune response of animals during the first days of infection. According to our results, for systemic infection of bacteria, to explore and develop PK/PD model exactly, data from this kind of animal experiment, specifically bacterial loading reduction in different organs as PD endpoints, should be considered. The knowledge gained in this kind of study is important for the validation of PK/PD models and is critical to predict clinical efficacy and rational dosage recommendation. This study had some limitations. First, only one bacterium was evaluated; to confirm our findings, other bacteria in different backgrounds and from different species should be studied. Second, because of a lack of MIC data, we could not recommend the rational dose of danofloxacin against *S. typhimurium*. Further research is essential to determine the rational usage of danofloxacin.

## Conclusion

*In vivo* PK/PD modeling of danofloxacin against *S. typhimurium* in rabbits using bacterial loading in four organs (blood, liver, spleen, and lung) as PD endpoints was conducted. The AUC_24 h_/MIC correlated well with the *in vivo* antibacterial effectiveness in the different organs, illustrating that the selection of the organ for PD determination had little effect on best PK/PD parameter determination. The AUC_24 h_/MIC ratios for bactericidal effect were 121.30, 354.28, 216.64, and 228.66 in blood, liver, spleen, and lung, respectively, indicating that the selection of target organ for PD determination is critical for parameter magnitude calculation in antimicrobial PK/PD modeling, and in addition, would affect a rational dosage optimization process.

## Author Contributions

XX and Z-QW designed this study and revised and guided the experiment. XX wrote this manuscript and participated in the whole experiment process. LP managed the whole experiment and analyzed the data. L-JJ and W-XL participated in all the experiments. J-YX and Y-JJ helped with the sampling process and concentration detection.

## Conflict of Interest Statement

The authors declare that the research was conducted in the absence of any commercial or financial relationships that could be construed as a potential conflict of interest.

## Supplementary Material

FIGURE S1 Chromatograms of blank rabbit serum **(A)** and blank rabbit serum spiked at the level of 0.02 g/mL **(B)**.
