# Supplementary figures and images for: In Vivo Pharmacokinetic/Pharmacodynamic Profiles of Danofloxacin in Rabbits Infected With Salmonella typhimurium After Oral Administration
Source: Front Pharmacol. 2018 Apr 17;9:391. doi: 10.3389/fphar.2018.00391 (PMC5913287; doi:10.3389/fphar.2018.00391)

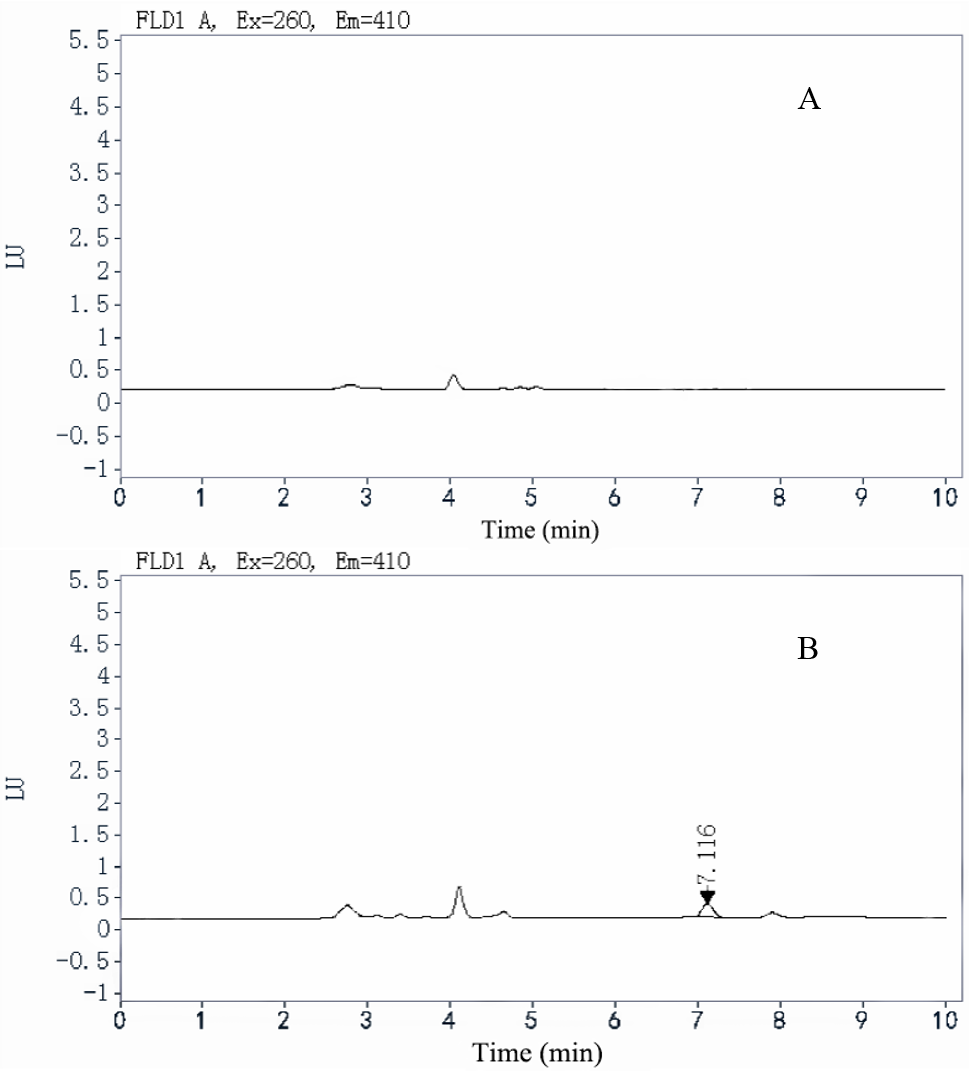

Supplement: FIGURE S1 — Chromatograms of blank rabbit serum (A) and blank rabbit serum spiked at the level of 0.02 g/mL (B). [file Image_1.TIF]
